# Modification effect of changes in cardiometabolic traits in association between kidney stones and cardiovascular events

**DOI:** 10.3389/fcvm.2022.923981

**Published:** 2022-07-26

**Authors:** Min Xu, Zhiyun Zhao, Feixia Shen, Ruying Hu, Jieli Lu, Yu Xu, Tiange Wang, Mian Li, Gang Chen, Li Chen, Lulu Chen, Yuhong Chen, Huacong Deng, Zhengnan Gao, Yanan Huo, Qiang Li, Chao Liu, Zuojie Luo, Yiming Mu, Guijun Qin, Yingfen Qin, Lixin Shi, Qing Su, Qin Wan, Guixia Wang, Shuangyuan Wang, Youmin Wang, Shengli Wu, Yiping Xu, Li Yan, Tao Yang, Zhen Ye, Xuefeng Yu, Yinfei Zhang, Jiajun Zhao, Tianshu Zeng, Weiqing Wang, Yufang Bi, Xulei Tang, Guang Ning

**Affiliations:** ^1^Department of Endocrine and Metabolic Diseases, Ruijin Hospital, Shanghai Institute of Endocrine and Metabolic Diseases, Shanghai Jiao Tong University School of Medicine, Shanghai, China; ^2^Shanghai National Clinical Research Center for Metabolic Diseases, Key Laboratory for Endocrine and Metabolic Diseases of the National Health Commission of the PR China, Shanghai Key Laboratory for Endocrine Tumor, State Key Laboratory of Medical Genomics, Ruijin Hospital, Shanghai Jiao Tong University School of Medicine, Shanghai, China; ^3^Department of Endocrinology, The First Affiliated Hospital of Wenzhou Medical University, Wenzhou, China; ^4^Institute of Chronic Disease, Zhejiang Provincial Center for Disease Control and Prevention, Hangzhou, China; ^5^Department of Endocrinology, Fujian Provincial Hospital, Fujian Medical University, Fuzhou, China; ^6^Department of Endocrinology, Qilu Hospital of Shandong University, Jinan, China; ^7^Department of Endocrinology, Union Hospital, Tongji Medical College, Huazhong University of Science and Technology, Wuhan, China; ^8^Department of Endocrinology, The First Affiliated Hospital of Chongqing Medical University, Chongqing, China; ^9^Department of Endocrinology, Dalian Municipal Central Hospital, Dalian, China; ^10^Department of Endocrinology, Jiangxi Provincial People's Hospital Affiliated to Nanchang University, Nanchang, China; ^11^Department of Endocrinology, The Second Affiliated Hospital of Harbin Medical University, Harbin, China; ^12^Department of Endocrinology, Jiangsu Province Hospital on Integration of Chinese and Western Medicine, Nanjing, China; ^13^Department of Endocrinology, The First Affiliated Hospital of Guangxi Medical University, Nanning, China; ^14^Department of Endocrinology, Chinese People's Liberation Army General Hospital, Beijing, China; ^15^Department of Endocrinology, The First Affiliated Hospital of Zhengzhou University, Zhengzhou, China; ^16^Department of Endocrinology, The First Affiliated Hospital of Guangxi Medical University, Nanning, China; ^17^Department of Endocrinology, Affiliated Hospital of Guiyang Medical College, Guiyang, China; ^18^Department of Endocrinology, Xinhua Hospital Affiliated to Shanghai Jiao Tong University School of Medicine, Shanghai, China; ^19^Department of Endocrinology, The Affiliated Hospital of Southwest Medical University, Luzhou, China; ^20^Department of Endocrinology, The First Hospital of Jilin University, Changchun, China; ^21^Department of Endocrinology, The First Affiliated Hospital of Anhui Medical University, Hefei, China; ^22^Department of Endocrinology, Karamay Municipal People's Hospital, Xinjiang, China; ^23^Clinical Trials Center, Ruijin Hospital Affiliated to Shanghai Jiao Tong University School of Medicine, Shanghai, China; ^24^Department of Endocrinology, Sun Yat-sen Memorial Hospital, Sun Yat-sen University, Guangzhou, China; ^25^Department of Endocrinology, The First Affiliated Hospital of Nanjing Medical University, Nanjing, China; ^26^Department of Endocrinology, Tongji Hospital, Tongji Medical College, Huazhong University of Science and Technology, Wuhan, China; ^27^Department of Endocrinology, Central Hospital of Shanghai Jiading District, Shanghai, China; ^28^Department of Endocrinology, Shandong Provincial Hospital Affiliated to Shandong University, Jinan, China; ^29^Department of Endocrinology, The First Hospital of Lanzhou University, Lanzhou, China

**Keywords:** kidney stone, CVD, metabolic disorders, modification effect, longitudinal change

## Abstract

**Backgrounds:**

Whether longitudinal changes in metabolic status influence the effect of kidney stones on cardiovascular disease (CVD) remains unclarified. We investigated the modification effect of status changes in metabolic syndrome (MetS) in the association of kidney stones with risk of incident CVD events.

**Methods:**

We performed a prospective association and interaction study in a nationwide cohort including 129,172 participants aged ≥ 40 years without CVDs at baseline and followed up for an average of 3.8 years. Kidney stones information was collected by using a questionnaire and validated by medical records. The repeated biochemical measurements were performed to ascertain the metabolic status at both baseline and follow-up.

**Results:**

4,017 incident total CVDs, 1,413 coronary heart diseases (CHDs) and 2,682 strokes were documented and ascertained during follow-up. Kidney stones presence was significantly associated with 44%, 70% and 31% higher risk of CVDs, CHDs and stroke, respectively. The stratified analysis showed significant associations were found in the incident and sustained MetS patients, while no significant associations were found in the non-MetS at both baseline and follow-up subjects or the MetS remission ones, especially in women. For the change status of each single component of the MetS, though the trends were not always the same, the associations with CVD were consistently significant in those with sustained metabolic disorders, except for the sustained high blood glucose group, while the associations were consistently significant in those with incident metabolic disorders except for the incident blood pressure group. We also found a significant association of kidney stone and CVD or CHD risk in the remain normal glucose or triglycerides groups; while the associations were consistently significant in those with incident metabolic disorders except for the incident blood pressure group. We also found a significant association of kidney stone and CVD or CHD risk in the remain normal glucose or triglycerides groups.

**Conclusions:**

A history of kidney stones in women with newly developed MetS or long-standing MetS associated with increased risk of CVD. The mechanisms link kidney stones and CVD risk in the metabolic and non-metabolic pathways were warranted for further studies.

## Introduction

Compelling data showed that kidney stones independently associated with a higher risk of cardiovascular diseases (CVDs), including coronary heart diseases (CHD) and stroke in large cohort studies and meta-analysis of cohort studies (1–6). Kidney stones, also known as nephrolithiasis, was recognized as a systemic disorder and closely associated with many cardiometabolic diseases such as diabetes, obesity, hypertension, metabolic syndrome (MetS) (7–9) and chronic kidney disease (10). However, whether the dynamic changes in metabolic status possess effect on the association of kidney stones with the risk of CVDs remains unexplored.

An increased risk of myocardial infarction was found in women with kidney stones but not in men (1, 3, 5). While in most studies, the prevalence and risk of forming the kidney stones and CVDs is higher in men than that in women. Whether the sex difference of the risk of CVDs in relation to kidney stone needs to be confirmed. The forming of kidney stones and CVDs shared many lifestyle factors, such as old age and unhealthy diet. It has been suggested that lifestyle modifications, such as weight loss, diet improvement, smoking cessation and exercise, will help prevent the development of both kidney stones and vascular disease. However, less evidence is provided.

Both the prevalence of kidney stones and CVDs increased and varies across countries (11–13). According to National Health and Nutrition Examination Survey, the prevalence of kidney stones is 10.6% in men and 7.1% in women, and the number continues to rise (11, 12). In China, the kidney stone prevalence was from 5.95% to 10.63% from the year 1991 to date (13). Kidney stones can cause significant morbidity including urinary tract infection, flank pain, hydronephrosis, decreased renal function, etc. Due to lack of satisfactory therapeutic options and a high recurrence rate of up to 50% within the subsequent 5–10 years after the first episode, the rate of repeated operative intervention is high. Giving the increasing rate of kidney stones and CVDs and the heavy health burden they bring, identifying the modifiers of associations of kidney stones and risk of CVDs is extremely important for high-risk stratification and precision prevention. In the present study, we aimed to investigate primarily the modification effect of changes in metabolic status on the association between kidney stones and risk of incident CVDs in a large, nation-wide, prospective Chinese cohort; secondarily, the modification effect of sex and lifestyle factors on the associations. To clarify the effect of dynamic changes in metabolic status in the association of kidney stones with the risk of CVDs would be helpful to identify the specific and better management of individuals with kidney stones and at a high risk of CVDs.

## Methods

### Study population

The study participants were from an ongoing multicenter, population-based cohort study, the China Cardiometabolic Disease and Cancer Cohort (4C) Study (14, 15). Briefly, during 2011–2012, a total of 20 communities from various geographic regions in China were selected. The eligible men and women aged ≥ 40 years were invited to the study by home visits by the trained community health workers. At baseline, we used a questionnaire to collect the lifestyle factors, disease and medical history, etc. We also performed anthropometry measurements, 2-h oral glucose tolerance tests (OGTTs), blood and urine sampling. During 2014–2015, we conducted the first round of follow-up examinations.

We recruited a total of 193,846 individuals at the baseline and 170,240 were invited and participated in the follow-up. We excluded the baseline CVDs or cancers (*n* = 2,826) or other kidney diseases (*n* = 1,666), or those were missing information on kidney stones (*n* = 13,974), or who failed to be followed up for collecting the information on CVD events (*n* = 22,602). Thus, a total of 129,172 participants at baseline were finally included in the analysis for risk of CVDs (Supplementary Figure 1) (16).

### Ascertainment of cardiovascular events

The incident CVD events were the composite of fatal or non-fatal myocardial infarction or stroke, and hospitalized or treated heart failure. The fatal or non-fatal myocardial infarction was also defined as CHD. Myocardial infarction was defined as changes in troponin T and creatine-kinase-MB isoform levels, or in electrocardiogram results, or with symptoms of myocardial ischemia. Stroke was defined as a fixed neurological deficit at least 24 h because of a presumed vascular cause. Heart failure was defined as a clinical syndrome presenting with multiple signs and symptoms consistent with cardiac decompensation or inadequate cardiac pump function. Deaths and clinical outcomes were collected from local vital registries of the National Disease Surveillance Point System and the National Health Insurance System. The Clinical Outcome Adjudication Committee is from Ruijin Hospital, Shanghai Jiao Tong University School of Medicine and composed of ten unbiased experts in cardiology, oncology, neurology, and endocrine and metabolic diseases. All members of the committee were unaware of baseline risk factors of study participants. Two members of the committee were assigned potential causes of mortality according to a standard clinical outcome adjudication procedure and independently verified each clinical event. Discrepancies were adjudicated by discussions involving other members of the committee.

In the time-to-event analysis, participants were censored at the date of CVD diagnosis, death, or the end of follow-up, whichever occurred first. Person-years was calculated from the enrollment date to the censoring date.

### Data on kidney stones

Questions about the history of kidney stones were asked in the baseline questionnaires. History of kidney stones was defined as self-reported presence of kidney stones or nephrolithiasis (ICD-10: N20.000), or receiving a procedure of percutaneous nephrolithotomy (PCNL) (ICD-9-CM-3: 55.0402). Participants reporting a history of kidney stones were asked about the date of occurrence, the diagnosed hospital and the tests or procedures they were advised to perform. The self-reported diagnosis was confirmed by the medical records.

### Data collection

We used questionnaires to collect demographic characteristics, dietary and lifestyle factors, family history, and medical history by face-to-face interviews. Smoking status was categorized into current, former and never smoking. Average alcohol consumption was calculated by multiplying the amount of alcohol consumed per drinking day by frequency (g/day). Education attainment was classified as high school and above or less. International Physical Activity Questionnaire was used to assess physical activity. A food frequency questionnaire was used to collect habitual dietary intake by asking the consumption frequency and portion size of typical food items during the previous 12 months (17). According to the 2020 American Heart Association (AHA) Strategic Impact Goals (18), the healthy diet was defined as the follows, 1) the fruit and vegetable intake of at least 4.5 cups/day, 2) the carbonated beverage <450 Kal per day, 3) the red meat consumption <50 g per day, 4) the bean consumption greater equal 25 g per day, and 5) the fish intake greater or equal 7.0 g per day. We assigned each healthy diet habits as 1, without the healthy habit as 0. We summed the total score of all the 5 healthy diet habit as the dietary score.

Body mass index (BMI) was calculated as body weight (kg) divided by the height (squared meters). Waist circumference was measured at the midway between the lower edge of the costal arch and the upper edge of the iliac crest. Three measurements of systolic and diastolic blood pressure (SBP and DBP) were obtained by using an automated electronic device (OMRON Model HEM-752 FUZZY, Dalian, China) in a seated position after at least a 5-min rest, and the average of the 3 readings were used for analysis.

All participants underwent a 2-h, 75-gram OGTT after an overnight fast of at least 10 h. Fasting and 2-h plasma glucose concentrations were measured locally using a glucose oxidase or hexokinase method. Serum insulin, low- and high-density lipoprotein (LDL and HDL) cholesterol, triglycerides, and serum creatinine were measured at the central laboratory using an auto-analyzer (ARCHITECT ci16200, Abbott Laboratories, Chicago, IL, USA). Insulin resistance was estimated by the homeostasis model assessment of insulin resistance (HOMA-IR) index: fasting insulin (μIU/mL) × fasting glucose (mmol/L) /22.5. Estimated glomerular filtration rate (eGFR) was calculated using the Chronic Kidney Disease Epidemiology Collaboration equation (19).

### Definitions

MetS was defined according to the revised National Cholesterol Education Program/Adult Treatment Panel III (NCEP/ATP III) criteria (2004) (20), when three or more of the following criteria were met: 1) blood pressure ≥ 130/85 mmHg or taking antihypertensive drugs, 2) waist circumference ≥ 90 cm in men and ≥ 80 cm in women, 3) triglycerides ≥ 1.69 mmol/L, 4) HDL cholesterol <1.03 mmol/L in men and <1.29 mmol/L in women, 5) fasting plasma glucose ≥ 5.6 mmol/L or taking hypoglycemic medications. The missing values for each metabolic trait were imputed to the mean values for normal distribution variables and the median values for skewed distribution variables.

The change status of the MetS and its components was grouped into four categories: 1) remain no MetS/or each component at both baseline and follow-up examinations, 2) incident MetS/or each component during follow-up, 3) remission of MetS/or each component from baseline to follow-up, and 4) sustained MetS/or each component from baseline to follow-up.

### Statistical analysis

Baseline characteristics of participants were shown as means with standard deviations (SDs), or medians (interquartile range) for continuous variables and numbers with percentages for categorical variables. Cox proportional hazards models were used to calculate hazard ratios (HRs) and 95% confidence intervals (CIs) for CVD events associated with baseline kidney stones in total participants, men and women, and in each category of change status of MetS, with adjustments for traditional CVD risk factors, age (years), baseline BMI (kg/m^2^), waist circumference (cm), quartiles of physical activity, quartiles of sedentary time, smoking status (current, former and never), alcohol drinking (g/day), and education level (high school and above, or less), SBP and DBP (mmHg), fasting and OGTT 2-h glucose (mmol/L), HOMA-IR, LDL and HDL cholesterol (mmol/L), triglycerides (mmol/L), gall stone (yes or no), diet score and eGFR. If covariate information was missing, we imputed the mean values for continuous variables or used a missing indicator for categorical variables. Multiplicative interaction effects of kidney stones and the interested cardiometabolic risk factors or lifestyles on CVD events were tested by including the product term, e.g., MetS category × kidney stones (yes or no), the MetS category, kidney stones (yes or no), and the covariates in the models simultaneously. The covariates were same as above used in the main analysis. Linear regression models with generalized estimating equations (GEE) were used to examine the association of baseline kidney stones with two time-points (baseline and follow-up) measures of the cardiometabolic traits. Statistical significance was assessed at a two-sided *P*-value of <0.05 by using the SAS software, version 9.4 (SAS Institute Inc).

## Results

The 129,172 adults (44,958 men and 84,214 women) aged ≥ 40 years were followed up for an average of 3.8 years. The presence rate of kidney stones was 4.28% in men and 2.68% in women (Table 1). A total of 4,017 incident CVD events were identified after 579,373 person-years of follow-up.

**Table 1:** Baseline characteristics of study population according to kidney stones presence status by sex.

**Variables**	**Total**			**Men**			**Women**		
	**Kidney stones**, **no**	**Kidney stones**, **yes**	***P***	**Kidney stones**, **no**	**Kidney stones**, **yes**	***P***	**Kidney stones**, **no**	**Kidney stones**, **yes**	***P***
n	124,992	4,180		43,035	1,923		81,957	2,257	
Age, year	56.8 ± 9.1	56.7 ± 8.9	0.60	57.7 ± 9.4	56.8 ± 9.18	< .0001	56.3 ± 8.9	56.6 ± 8.6	0.07
Body mass index, kg/m^2^	24.6 ± 3.6	24.7 ± 3.5	0.84	24.8 ± 3.5	24.9 ± 3.4	0.13	24.6 ± 3.6	24.4 ± 3.6	0.09
Waist circumference, cm	84.1 ± 9.8	84.8 ± 9.6	< .0001	86.7 ± 9.6	87.1 ± 9.3	0.07	82.7 ± 9.7	82.9 ± 9.4	0.45
Systolic blood pressure, mmHg	133 ± 21	133 ± 20	0.23	136 ± 20	134 ± 20	0.002	132 ± 21	132 ± 21	0.44
Diastolic blood pressure, mmHg	78 ± 11	79 ± 11	0.0002	81 ± 11	81 ± 11	0.77	77 ± 11	78 ± 11	0.08
Fasting plasma glucose, mmol/l	5.96 ± 1.63	5.95 ± 1.53	0.71	6.12 ± 1.79	6.05 ± 1.65	0.08	5.87 ± 1.54	5.86 ± 1.43	0.79
OGTT 2h plasma glucose, mmol/L	8.25 ± 3.84	8.47 ± 3.81	0.0002	8.37 ± 4.16	8.51 ± 4.04	0.16	8.18 ± 3.66	8.44 ± 3.62	0.0008
HOMA–IR	1.71 (1.17–2.56)	1.79 (1.22–2.64)	0.003	1.57 (1.02–2.42)	1.71 (1.11–2.54)	0.27	1.80 (1.25–2.64)	1.84 (1.29–2.74)	0.0002
HDL cholesterol, mmol/l	1.33 ± 0.36	1.30 ± 0.36	< .0001	1.26 ± 0.36	1.22 ± 0.36	< .0001	1.37 ± 0.35	1.36 ± 0.35	0.07
LDL cholesterol, mmol/l	2.88 ± 0.88	2.88 ± 0.89	0.85	2.79 ± 0.84	2.81 ± 0.86	0.40	2.93 ± 0.89	2.95 ± 0.91	0.29
Total cholesterol, mmol/l	4.96 ± 1.14	4.96 ± 1.17	0.78	4.80 ± 1.10	4.81 ±1.14	0.78	5.05 ± 1.15	5.09 ± 1.18	0.12
Triglycerides, mmol/l	1.32 (0.94–1.92)	1.36 (0.97–2.00)	0.0003	1.33 (0.93–2.00)	1.71 (1.11–2.52)	0.01	1.31 (0.94–1.88)	1.35 (0.97–1.95)	0.14
Alcohol consumption, g/day	46.7 ± 174.0	60.6 ± 206.7	< .0001	128.9 ± 274.2	127.7 ± 289.2	0.85	3.82 ± 38.72	3.98 ± 35.46	0.84
Physical activity (METS–h/wk)	30.6 ±40.6	32.4 ±41.4	0.005	31.3 ± 43.7	31.8 ± 43.6	0.67	30.2 ± 38.8	32.9 ± 39.3	0.001

Data are presented as means ± standard deviation (SD), or medians (inter–quartile ranges) for skewed variables, or number (proportions) for categorical variables. MET, metabolic equivalent task; HOMA–IR indicates homeostasis model assessment of insulin resistance. HDL cholesterol, high–density lipoprotein cholesterol; LDL cholesterol, low–density lipoprotein cholesterol.

**Figure 1:**
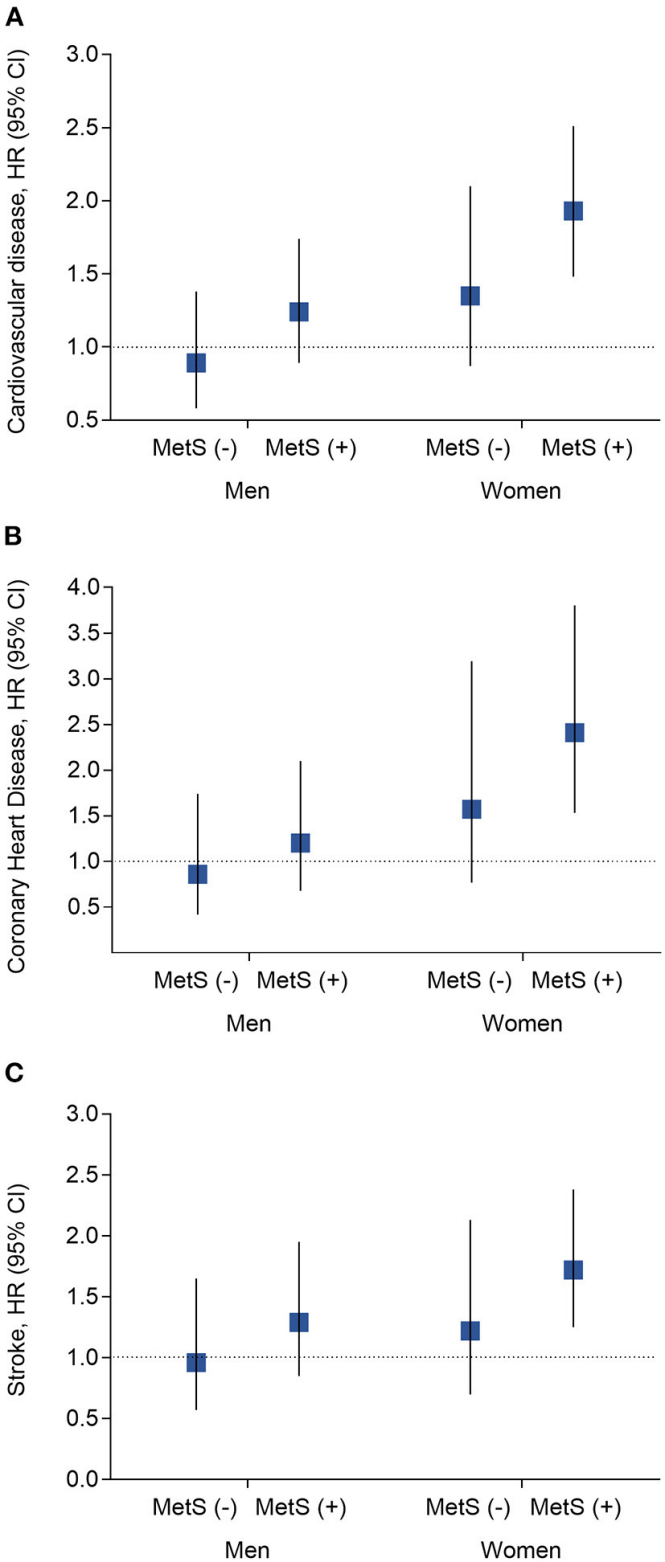
Analysis of the hazard risk of kidney stones with risk of incident cardiovascular events by MetS x sex interaction subgroups. **(A)** Risk of cardiovascular diseases; **(B)** Risk of coronary heart disease; **(C)** Risk of stroke. Data are present as hazard ratio (HR) and 95% confidence interval (CI). *P*-values were calculated from the multivariable Cox regression models. Adjustments included for age (year), baseline level of body mass index (kg/m^2^), waist circumference (cm), quartiles of physical activity, quartiles of sedentary time, smoking status (current, former, and never), alcohol drinking (g/l), education level (percentage of high school and above), systolic and diastolic blood pressure (mmHg), fasting plasma glucose (mmol/l), oral glucose tolerance test 2-h glucose (mmol/l), HOMA-IR, low- and high-density lipoprotein cholesterol (mmol/l), and triglycerides (mmol/l), gall stone (yes or no), diet score and eGFR. MetS, metabolic syndrome.

**Figure 2:**
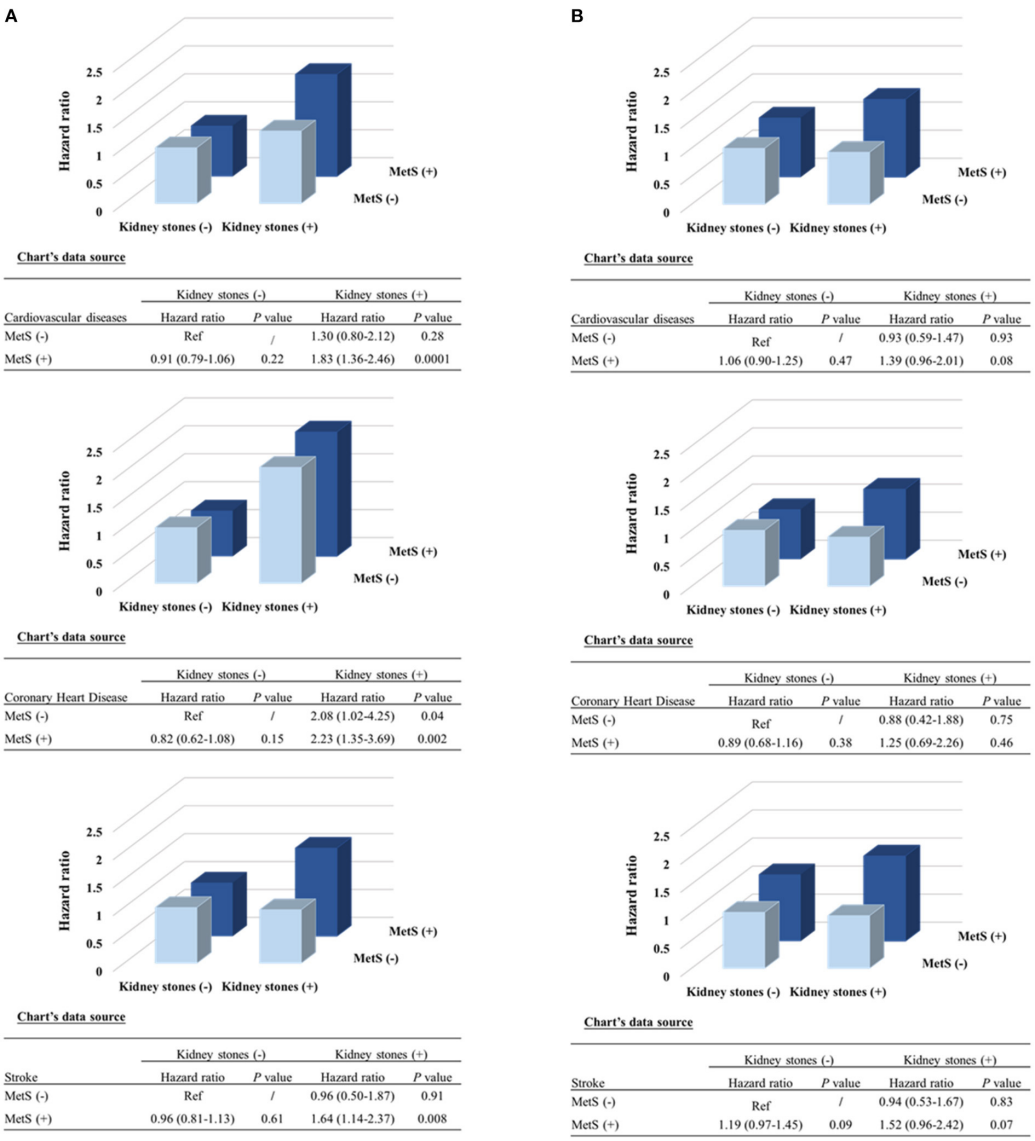
Combined effect of kidney stones and MetS presence on risk of incident cardiovascular events by sex. **(A)** Women; **(B)** Men. *P*-values were calculated from the multivariable Cox regression models, after adjustments for the same covariates as Figure 1. MetS, metabolic syndrome.

The presence of kidney stones was associated with a 44% high risk of total CVDs (95% CI, 1.20–1.72; *P* = 0.0001), 70% of CHDs (95% CI, 1.27–2.28; *P* = 0.0004) and 31% of stroke (95% CI, 1.04–1.64; *P* = 0.02). The associations were more prominent in women (the adjusted HR is 1.77 for CVDs [*P* < 0.0001], 2.50 for CHD [*P* < 0.0001], and 1.48 for stroke [*P* = 0.01]); while no significant associations were found in men (all *P* > 0.35), after adjustments for the conventional risk factors (Table 2). The association was also stronger in participants with MetS (HR 1.66) than that without MetS (HR 1.12) (*P* for interaction =0.0006) (Supplementary Table 1) (16). We further stratified the participants according to both sex and baseline MetS status, as compared to participants without kidney stones, those with kidney stones was with the most significant higher risk of CVD events in women with MetS (Figure 1). The joint effect analysis showed that in women, as compared to those without MetS or kidney stones, participants with both MetS and kidney stones possessed the highest HRs for incident risk of CVD events (Figure 2A); while in men, the combine effect was not significant either (Figure 2B). The associations were nominally stronger in central obesity, hypertension, dyslipidemia and lower eGFR than those without these disorders (Supplementary Table 1) (16).

**Table 2:** Hazard risk of kidney stones with risk of incident cardiovascular events.

	**No. of cases**	**Person–years**	**Incidence rate, %**	**Multivariable–adjusted HR (95% CI)**					
				**Model 1**	***P1***	**Model 2**	***P2***	**Mode 3**	***P3***
**Total**									
**Cardiovascular disease**									
Kidney stones, no	3,849	560,629	3.08	1 (Reference)		1 (Reference)		1 (Reference)	
Kidney stones, yes	168	18,744	4.02	1.29 (1.11–1.51)	0.001	1.31 (1.12–1.55)	0.001	1.44 (1.20–1.72)	0.0001
**Coronary heart disease**									
Kidney stones, no	1,353	564,003	1.08	1 (Reference)		1 (Reference)		1 (Reference)	
Kidney stones, yes	60	18,883	1.44	1.33 (1.02–1.72)	0.03	1.31 (0.99–1.72)	0.06	1.70 (1.27–2.28)	0.0004
**Stroke**									
Kidney stones, no	2,569	561,644	2.06	1 (Reference)		1 (Reference)		1 (Reference)	
Kidney stones, yes	113	18,804	2.70	1.30 (1.07–1.57)	0.008	1.34 (1.10–1.64)	0.003	1.31 (1.04–1.64)	0.02
**Men**									
**Cardiovascular disease**									
Kidney stones, no	1,717	190,565	3.99	1 (Reference)		1 (Reference)		1 (Reference)	
Kidney stones, yes	75	8,545	3.90	1.05 (0.83–1.32)	0.69	1.02 (0.79–1.31)	0.88	1.14 (0.86–1.51)	0.35
**Coronary heart disease**									
Kidney stones, no	672	192,004	1.56	1 (Reference)		1 (Reference)		1 (Reference)	
Kidney stones, yes	29	8,605	1.51	1.06 (0.73–1.54)	0.77	0.97 (0.65–1.46)	0.89	1.16 (0.74–1.83)	0.51
**Stroke**									
Kidney stones, no	1,078	191,075	2.50	1 (Reference)		1 (Reference)		1 (Reference)	
Kidney stones, yes	49	8,573	2.55	1.08 (0.81–1.44)	0.62	1.10 (0.81–1.49)	0.56	1.14 (0.80–1.61)	0.48
**Women**									
**Cardiovascular disease**									
Kidney stones, no	2,132	370,065	2.60	1 (Reference)		1 (Reference)		1 (Reference)	
Kidney stones, yes	93	10,198	4.12	1.59 (1.29–1.96)	< .0001	1.66 (1.34–2.06)	< .0001	1.77 (1.40–2.25)	< .0001
**Coronary heart disease**									
Kidney stones, no	681	371,999	0.83	1 (Reference)		1 (Reference)		1 (Reference)	
Kidney stones, yes	31	10,278	1.37	1.73 (1.21–2.48)	0.003	1.81 (1.25–2.63)	0.001	2.50 (1.70–3.67)	< .0001
**Stroke**									
Kidney stones, no	1,491	370,569	1.82	1 (Reference)		1 (Reference)		1 (Reference)	
Kidney stones, yes	64	10,231	2.84	1.53 (1.19–1.97)	0.001	1.60 (1.23–2.07)	0.0004	1.48 (1.09–2.00)	0.01

Data are hazard ratio (HR), 95% confidence interval (CI). P–values were calculated from the Cox regression models. Model 1, adjusted for age (years), sex, body mass index (kg/m^2^), waist circumference (cm); Model 2, further adjusted for quartiles of physical activity, quartiles of sedentary time, smoking status (current, former and never), alcohol drinking (g/day), and education level (high school and above, or less), based on Model 1; Model 3, further adjusted for systolic and diastolic blood pressure (mmHg), fasting plasma glucose (mmol/L), oral glucose tolerance test (OGTT) 2–h glucose (mmol/L), HOMA–IR, low– and high–density lipoprotein cholesterol (mmol/L), triglycerides (mmol/L), gall stone (yes or no), diet score, and eGFR, based on Model 2. P for interaction of sex is 0.02 for cardiovascular disease events, 0.01 for coronary heart disease, and 0.27 for stroke.

The distribution of change status of the MetS and its components were shown in Table 3, Supplementary Figure 2, and Supplementary Table 2 (16). Kidney stones at baseline were significantly associated with a higher risk of incident CVDs and CHDs in the incident MetS group (HR 2.31, 95%CI 1.28–4.18, *P* = 0.005 for CVD; HR 3.62, 95% CI 1.41–9.27, *P* =0.007 for CHD) and the sustained MetS group (HR 1.58, 95%CI 1.13–2.22, *P*= 0.01 for CVD; and HR 2.16, 95% CI 1.22–3.82, *P* =0.008 for CHD). No significant associations were found in the non-MetS at both baseline and follow-up group (HR 1.33, 95%CI 0.79–2.24 for CVD and 1.67 (0.77–3.64) for CHD, both *P* ≥ 0.20), or the MetS remission group (HR 1.75, 95%CI 0.91–3.35 for CVD and 1.57 (0.37–6.68) for CHD both *P* ≥ 0.09). We did not detect a significant association of kidney stones with risk of stroke in each category of the change status of MetS (all *P* ≥0.12). For the change status of the component of MetS, the trends were not always the same. However, the associations with CVDs were consistently significant in those with sustained metabolic disorders, except for the sustained high blood glucose group; while the associations were consistently significant in those with incident metabolic disorders except for the incident blood pressure group. We also found a significant association of kidney stone and CVD or CHD risk in the remain normal glucose or triglycerides groups. In addition, the association of kidney stone with risk of stroke was significant in the sustained high blood pressure, low HDL_c and high triglycerides group (Table 3). The results were consistently confirmed in women (Supplementary Tables 3, 4) (16).

**Table 3:** Hazard risk of kidney stones with risk of incident cardiovascular events by status changes in metabolic syndrome from baseline to follow–up.

	**Cardiovascular disease**		**Coronary heart disease**		**Stroke**	
	**HR (95% CI)**	***P*** **-value**	**HR (95% CI)**	***P*** **-value**	**HR (95% CI)**	***P*** **–value**
**MetS status changes**					
Remain no MetS						
Cases/participants	556/36,578		109/26,838		238/26,838	
Multivariable adjusted model	1.33 (0.79–2.24)	0.28	1.67 (0.77–3.64)	0.20	1.07 (0.53–2.18)	0.84
Incident MetS						
Cases/participants	284/12,258		49/8,624		129/8,624	
Multivariable adjusted model	2.31 (1.28–4.18)	0.005	3.62 (1.41–9.27)	0.007	1.80 (0.84–3.88)	0.13
MetS remission						
Cases/participants	216/10,097		35/7,775		117/7,775	
Multivariable adjusted model	1.75 (0.91–3.35)	0.09	1.57 (0.37–6.68)	0.54	1.75 (0.85–3.63)	0.13
Sustained MetS						
Cases/participants	1,022/31,440		182/23,907		528/23,907	
Multivariable adjusted model	1.58 (1.13–2.22)	0.01	2.16 (1.22–3.82)	0.008	1.39 (0.92–2.10)	0.12
**Central obesity status changes**						
Remain no central obesity						
Cases/participants	556/30,330		90/22,085		238/22,085	
Multivariable adjusted model	1.41 (0.86–2.31)	0.17	2.38 (1.14–4.97)	0.02	1.03 (0.53–2.02)	0.92
Incident central obesity						
Cases/participants	259/12,699		44/9,087		124/9,087	
Multivariable adjusted model	2.86 (1.61–5.08)	0.0003	3.51 (1.24–9.94)	0.02	2.83 (1.47–5.45)	0.002
Central obesity remission						
Cases/participants	193/8,152		35/6,103		93/6,103	
Multivariable adjusted model	1.31 (0.57–3.00)	0.52	0	0.99	1.97 (0.85–4.56)	0.12
Sustained central obesity						
Cases/participants	1125/40,837		212/30,424		565/30,424	
Multivariable adjusted model	1.62 (1.17–2.25)	0.004	2.30 (1.35–3.90)	0.002	1.30 (0.85–1.98)	0.22
**Blood pressure status changes**						
Remain normal blood pressure						
Cases/participants	222/23,442		50/18,302		100/18,302	
Multivariable adjusted model	2.13 (1.08–4.19)	0.03	1.86 (0.57–6.01)	0.30	2.18 (0.95–5.00)	0.07
Incident high blood pressure						
Cases/participants	182/11,235		37/8,217		83/8,217	
Multivariable adjusted model	0.78 (0.25–2.47)	0.67	0.85 (0.12–6.27)	0.88	0.77 (0.19–3.14)	0.71
High blood pressure remission						
Cases/participants	157/9,360		44/6,909		56/6,909	
Multivariable adjusted model	1.67 (0.67–4.14)	0.27	1.54 (0.37–6.42)	0.55	1.79 (0.55–5.82)	0.33
Sustained high blood pressure						
Cases/participants	1,639/49,935		259/35,027		791/35,027	
Multivariable adjusted model	1.71 (1.31–2.24)	0.0001	2.51 (1.61–3.90)	<0.0001	1.47 (1.06–2.03)	0.02
**Glucose metabolism status changes**						
Remain normal blood glucose						
Cases/participants	550/33,596		107/24,874		255/24,874	
Multivariable adjusted model	1.88 (1.22–2.91)	0.004	2.49 (1.25–4.98)	0.01	1.65 (0.96–2.83)	0.07
**Incident high blood glucose**						
Cases/participants	304/12,117		45/8,396		143/8,396	
Multivariable adjusted model	1.88 (0.98–3.58)	0.06	3.97 (1.53–10.3)	0.005	1.21 (0.49–2.97)	0.68
**Glucose metabolism remission**						
Cases/participants	223/13,430		36/9,846		109/9,846	
Multivariable adjusted model	1.67 (0.78–3.60)	0.19	2.69 (0.81–8.90)	0.10	1.30 (0.48–3.54)	0.61
Sustained high blood glucose						
Cases/participants	1,227/36,463		226/26,322		590/26,322	
Multivariable adjusted model	1.31 (0.95–1.80)	0.11	1.22 (0.66–2.24)	0.53	1.33 (0.91–1.94)	0.14
**HDL_c metabolism status changes**						
Remain normal HDL_c						
Cases/participants	937/47,604		156/32,951		414/32,951	
Multivariable adjusted model	1.07 (0.69–1.65)	0.78	1.38 (0.67–2.83)	0.38	0.97 (0.57–1.65)	0.90
Incident low HDL_c						
Cases/participants	433/12,115		74/8,199		184/8,199	
Multivariable adjusted model	1.85 (1.09–3.13)	0.02	2.69 (1.21–5.97)	0.01	1.44 (0.71–2.95)	0.32
HDL_c metabolism remission						
Cases/participants	221/13,084		44/10,725		128/10,725	
Multivariable adjusted model	2.04 (1.15–3.64)	0.02	2.14 (0.75–6.10)	0.16	1.91 (0.96–3.81)	0.07
Sustained low HDL_c						
Cases/participants	596/21,827		116/16,839		305/16,839	
Multivariable adjusted model	2.01 (1.35–3.00)	0.0006	2.41 (1.21–4.78)	0.01	1.94 (1.21–3.12)	0.006
**Triglycerides metabolism status changes**						
Remain normal triglycerides						
Cases/participants	694/35,747		193/35,747		517/35,747	
Multivariable adjusted model	1.77 (1.29–2.44)	0.0004	2.41 (1.44–4.04)	0.0008	1.45 (0.97–2.18)	0.07
Incident high triglycerides						
Cases/participants	185/9,898		43/9,898		144/9,898	
Multivariable adjusted model	0.85 (0.35–2.07)	0.72	1.48 (0.35–6.20)	0.59	0.66 (0.21–2.07)	0.48
Triglycerides metabolism remission						
Cases/participants	178/7,030		55/7,030		128/7,030	
Multivariable adjusted model	1.61 (0.84–3.09)	0.15	2.03 (0.71–5.77)	0.19	1.63 (0.75–3.53)	0.22
Sustained high triglycerides						
Cases/participants	333/16,033		99/16,033		242/16,033	
Multivariable adjusted model	1.66 (1.04–2.66)	0.03	1.63 (0.71–3.78)	0.25	1.73 (1.01–2.99)	0.048

Data are hazard ratio (HR), 95% confidence interval (CI). P–values were calculated from the Cox regression models. Model 1, adjusted for age (years), sex, body mass index (kg/m^2^), waist circumference (cm); Model 2, further adjusted for quartiles of physical activity, quartiles of sedentary time, smoking status (current, former and never), alcohol drinking (g/day), and education level (high school and above, or less), based on Model 1; Model 3, further adjusted for systolic and diastolic blood pressure (mmHg), fasting plasma glucose (mmol/L), oral glucose tolerance test (OGTT) 2–h glucose (mmol/L), HOMA–IR, low– and high–density lipoprotein cholesterol (mmol/L), triglycerides (mmol/L), gall stone (yes or no), diet score, and eGFR, based on Model 2.

The associations of kidney stones and CVD events were significant in participants who were younger than 65 years, never smoking, non-drinker, with a healthy diet score, and low level of physical activity (Supplementary Figure 3) (16).

We used the GEE models to investigate the presence of baseline kidney stones with the two time points of measurements of cardiovascular profiles (Supplementary Table 5). Baseline kidney stones were significantly associated with a higher level of LDL cholesterol, fasting insulin and HOMA_IR, which are cardio-risk factors, and a lower level of HDL cholesterol. The results were similar in men and women.

## Discussion

From the large nationwide longitudinal study in Chinese, the status changes in MetS and its components significantly modified the associations of kidney stones with the risk of incident CVD events. The presence of kidney stones was associated a higher risk of incident CVDs in the incident and the sustained MetS patients. No significant associations were found in the non-MetS at both baseline and follow-up subjects, or the MetS remission ones. The same trend was found for the risk of incident CHD, while we did not detect a significant association of kidney stones with risk of stroke in each category of the change status of MetS. For the change status of each single component of MetS, the trends were not always the same. However, the associations with CVD were consistently significant in those with sustained metabolic disorders, except for the sustained high blood glucose group; while the associations were consistently significant in those with incident metabolic disorders except for the incident blood pressure group. We also found a significant association of kidney stone and CVD or CHD risk in the remain normal glucose or triglycerides groups. In addition, the association of kidney stone with risk of stroke was significant in the sustained high blood pressure, low HDL_c and high triglycerides group. Our study provides the modifiers of associations of kidney stones with risk of CVDs, and evokes that more attention should be paid to women MetS patients with kidney stones, especially from better prevention and management of metabolic comorbidities and CVD risk point of view.

Kidney stones were found to be associated with a significantly higher risk of CVDs in Caucasians and the western countries. Though there is a considerable ethnicity difference in the prevalence of both kidney stones (12, 13, 21) and CVDs, the estimated risk of kidney stones with CVDs is similar (1, 3, 6). In a meta-analysis including 6 cohort studies that contained 49,597 patients with kidney stones and 3,558,053 controls, with 133,589 cardiovascular events, kidney stones were associated with an increased adjusted risk estimate for CHD (HR, 1.19; 95% CI, 1.05–1.35; *P* =0.05; *n* = 6 cohorts) and stroke (HR, 1.40; 95% CI, 1.20–1.64; *P* = 0.001; *n* = 3 cohorts). In particular, kidney stones conferred HRs of 1.29 (95% CI, 1.10–1.52; *n* = 6 cohorts) for myocardial infarction and 1.31 (95% CI, 1.05–1.65; *n* = 4 cohorts) for coronary revascularization, respectively. In Asians, the urinary calculi were independently associated with a higher risk of developing myocardial infarction (HR, 1.31), stroke (HR, 1.39), and total CVD events (HR, 1.38) from a propensity score-matched cohort study (4). Our results were similar but slightly higher as compared to the reported ones (the HRs for CVDs, CHD and stroke are 1.70, 1.31 and 1.44, respectively), which might mainly be due to different adjustments included. Thus, for the association of kidney stone with the risk of CVDs, our results were largely confirmatory and added evidence based on one large nation-wide Chinese cohorts.

Numerous data showed that the MetS and its components associated with both the forming of kidney stones and the risk of CVDs (22–25). Interestingly, the Alberta Kidney Disease Network reported (3), the associations of kidney stones with acute myocardial infarction events were more significant in subjects without diabetes, hypertension, cancers, or albuminuria. The study provided important stratification strata for the subjects with kidney stones to be cautious of the risk of developing CVDs. However, also there's other studies showed that the association was not significantly different in different age group, history of hypertension, diabetes, or Charlson Comorbidity Index Score (4). In our study, we found a stronger association of kidney stone with risk of CVD (P for interaction = 0.0006), CHD (P for interaction = 0.06) and stroke (P for interaction = 0.002) in those with metabolic syndrome at baseline. The associations were more prominent in those with central obesity, hypertension, dyslipidemia, and eGFR <90, though the interaction tests did not reach significant. The results were mostly consistent with what we found in the modification effect of the change status of the metabolic syndrome and each component of the metabolic syndrome.

Moreover, for the first time, we demonstrated that the longitudinal status changes in MetS and its components modified the associations of kidney stone with the risk of incident CVD events. The kidney stones were significantly associated higher risk of incident CVDs in the incident and the sustained MetS patients. We provided important evidence that those with MetS, especially without any remission of it, would be at much risk for future CVD events, while no significant association was found in those without MetS, even after adjusting for the confounders. We also found that in newly developed and sustained central obesity, newly developed and sustained low HDL cholesterol participants, the associations were stronger than that of without the metabolic disorders.

We did not find a significant modification effect of key lifestyle and dietary factors, such as smoking and drinking habits, physical activities, and healthy dietary habits on the associations of kidney stones and CVD. However, it showed nominally the association between CVD risk and kidney stones was more prominent in younger, never smoker, non-drinker, with healthy diet habit, and a high level of education. In most cases, the one-factor association was more pronounced when other cofounding was not existing. Take the above results in all, we speculated that this might be due to the absence of competing risk factors for CVDs in these relatively healthy subgroups, which is a common phenomenon in the epidemiological studies (3). When it comes to the development of the outcome disease and the change status of the co-existed metabolic disorders, in a more serious disease progression status, the risk of kidney stones, which were considered one of the systematic metabolic dysfunctions, might exert a higher risk for the CVDs risk. It is hard to give an exact explanation about the “contradiction”; however, this modification effect of the lifestyle factors or the other confounding factor at baseline and the longitudinal change in metabolic traits as well needs further investigation, especially to establish the pathophysiological basis of the associations and the modification effect.

We detected a significant sex difference in the association of kidney stone with risk of CVDs and the modification effect of the changes in metabolic status. The presence rate of kidney stones is higher in men than that in women. The incidence rate of CVD, CHD or stroke is higher in men than that in women (3.99% vs. 2.64% CVDs, 1.56% vs. 0.85% CHD, 2.51% vs. 1.85% stroke), too, as well as age, BMI, waist circumference, blood pressure, fasting and 2h-OGTT glucose, HDL-c, TG, and higher level of smoking, alcohol intake and physical activity. The data indicated that it is unlikely because of more metabolic disorders or risk factors in women, in fact, women are in a relatively healthy status.

However, we did find that as compared with men, more women were in sustained metabolic syndrome or incident metabolic syndrome (38.0% vs. 28.5%, and 13.7% and 13.3%, respectively); more sustained or incident central obesity (52.3% vs. 28.9, and 14.1% vs. 13.2%); more sustained or incident HDL-c (29.6% vs. 10.3%, and 14.5% vs. 9.1%); more sustained or incident high blood pressure (61.5% vs. 59.9%, and 12.3% vs. 11.8%) (Supplementary Table 2). It was consistent with that in the above sustained or incident metabolic disorders, the association of kidney stones with risk of CVDs was more prominent. We did not find a higher rate of incident or sustained high blood glucose in women than that in men. Similarly, we did not identify that the presence of kidney stones was associated a higher risk of incident CVDs in the incident and the sustained high blood glucose patients either.

However, we could not exclude the possibility that the difference was due to sex or women may be more likely exposed than men to unknown factors that could increase their cardiovascular and kidney stone risk, which need to be confirmed by further prospective studies. The mechanisms underlying this association also should be directed.

The forming of kidney stones and CVD shared many risk factors, such as obesity, hypertension, insulin resistance, and unhealthy dietary habits (26). The previous study discussed the potential mechanism for the occurs of kidney stones and CVD (27). Both diseases are chronic and characterized by accumulation of oxidized proteins and lipids in the renal tissue and arterial wall, respectively. On one hand, the dyslipidemia, perturbation of gut microbiome, obesity, high-fat diet, genetic factors, and infections produce higher oxalate, uric acid, calcium that leads to supersaturation of salts in urine. Increased reactive oxygen species (ROS) also enhances the crystal nucleation and aggregation it causes the crystal retention and the subsequent stone formation (27). On the other hand, during uric acid formation, ROS is formed from hypoxanthine. ROS causes the oxidation of LDL, and uric acid induces the TG biogenesis, both of which impair the endothelial dysfunction and lead to atherosclerosis (27). However, when we adjusted those shared factors, the kidney stones were still significantly associated with a higher risk of CVD. It may suggest pathway other than the known mechanism in the process of kidney stone formation and CVD events.

Among the several shared risk factors, insulin resistance was the common soil of the MetS, kidney stones and CVD risk, and oxidative stress might play an important role in the process from insulin resistance to endothelial cell dysfunction (28, 29). Dietary factors contributed to the forming kidney stones and increased risk of CVDs, such as high salt and sugar, low fruit and vegetables (26). It has been proposed taking actions in diet changes that are aimed at stone prevention (30) maybe at reduction of CVDs. The ideal cardiovascular health diet (18, 30) or the Mediterranean diet (31) was found not only better for prevention of CVDs, but also for prevention of kidney stone forming.

We have several strengths including the large nationwide sample size, the prospective study design, the repeated blood sampling and biochemical measurements, and the relatively full adjustments for potential confounders, and the well-validated adjudications of the incident CVD events. Limitations should be acknowledged. Firstly, the information on the presence of kidney stones was self-reported. It may underestimate the presence of kidney stones; however, the cases were validated by the medical records by the physician's diagnosis or the undergoing procedures. The recall bias could be minimized to the minimum. For one might not ignore the stone disease because of the obvious symptoms, such as pain, hematuria. Secondly, we could not determine the specific type of kidney stones. The most common types are made from calcium and oxalate, uric acid or cystine. Since uric acid stones are relatively rare in females (32), we speculated that calcium stones may be responsible for the associations. Thirdly, causality could not be determined between kidney stones and CVDs. The large bilateral Mendelian Randomization studies are needed to confirm our findings. Finally, we used the updated NCEP-ATPIII definitions for Asian-Americans. A previous study performed in a rural Chinese population showed that the Joint Interim Statement (JIS), International Diabetes Federation (IDF) and Chinese Diabetes Society (CDS) criteria may not be more suitable than the updated NCEP-ATPIII definitions, for screening high-risk individuals and estimating the risk of CHD and stroke from MetS (33). Thus, it would be acceptable for the present analysis using the updated NCEP-ATPIII definitions. Even though, the association of kidney stones, metabolic status, and CVDs in other age and ethnicity groups need further studies.

In conclusion, the present analysis suggest that a thorough cardiovascular assessment should be considered in patients who develop kidney stones especially in women, and in those with multi-metabolic disorders. Taking measures to prevent metabolic comorbidity may benefit both kidney and CVDs prevention, especially for those with both conditions.

## Data availability statement

The original contributions presented in the study are included in the article/Supplementary material, further inquiries can be directed to the corresponding authors.

## Ethics statement

The studies involving human participants were reviewed and approved by Medical Ethics Committee of Ruijin Hospital, Shanghai Jiao Tong University School of Medicine. The patients/participants provided their written informed consent to participate in this study.

## Author contributions

MX, ZZ, FS, RH, JL, and YuX: conceptualization, formal analysis, and writing–original draft. TW, ML, GC, LiC, LuC, YC, HD, ZG, YH, QL, CL, ZL, YM, GQ, YQ, LS, QS, QW, GW, SWa, YW, SWu, YiX, LY, TY, ZY, XY, YZ, JZ, and TZ: acquisition of data, revision of the manuscript for intellectual content. WW, YB, XT, and GN: supervision, funding acquisition, and revision of the manuscript for intellectual content. MX, WW, YB, XT, and GN: conceptualization, formal analysis, funding acquisition, and critical revision of the manuscript for important intellectual content. All authors contributed to the article and approved the submitted version.

## Funding

This work was supported by the National Natural Science Foundation of China (81941017, 81930021, 81970706, 81970728, 81870560, 82088102, 82022011, and 91857205), the Science and Technology Committee of Shanghai (20Y11905100) and the Clinical Research Plan of SHDC (SHDC2020CR1001A and SHDC2020CR3064B), and the Shanghai Municipal Education Commission–Gaofeng Clinical Medicine Grant Support (20152508 Round 2). MX, JL, ML, TW, YX, ZZ, YB, WW, and GN are members of innovative research team of high-level local universities in Shanghai.

## Conflict of interest

The authors declare that the research was conducted in the absence of any commercial or financial relationships that could be construed as a potential conflict of interest.

## Publisher's note

All claims expressed in this article are solely those of the authors and do not necessarily represent those of their affiliated organizations, or those of the publisher, the editors and the reviewers. Any product that may be evaluated in this article, or claim that may be made by its manufacturer, is not guaranteed or endorsed by the publisher.
