# Supplementary material for: Modification effect of changes in cardiometabolic traits in association between kidney stones and cardiovascular events
Source: Front Cardiovasc Med. 2022 Jul 26;9:923981. doi: 10.3389/fcvm.2022.923981 (PMC9360502; doi:10.3389/fcvm.2022.923981)
Supplement: Supplementary file 1 [file Data_Sheet_1.docx]

**Online-Only Supplementary Data**

Min Xu, Zhiyun Zhao, Feixia Shen et al: Modification effect of changes in cardiometabolic traits in the association between kidney stones and cardiovascular events

**Supplementary Figure 1.** Flow chart of the study participants.

**Supplementary Figure 2.** The prevalence of MetS, each of its components, and number of the components at baseline and follow up, respectively. A. The prevalence of MetS and each component. B. The number of the components. The light grey bars and the numbers on them indicate the prevalence at baseline. The blue bars and the numbers on them indicate the prevalence of the number of the components. The trend lines were added by using the 2 Period Moving Average (2 per. Mov. Avg) method. MetS, metabolic syndrome.

**Supplementary Figure 3.** Stratified analysis of the hazard risk of kidney stones with risk of incident cardiovascular events by age and lifestyle subgroups. A: Cardiovascular diseases; B: Coronary heart disease; C: Stroke. Data are present as hazard ratio (HR) and 95% confidence interval (CI). P values were calculated from the multivariable Cox regression models, after adjustments for the same covariates as Figure 1. MetS, metabolic syndrome.

**Supplementary Table 1.** Stratified analysis of the hazard risk of kidney stones with a risk of incident cardiovascular events by metabolic syndrome (MetS) status at baseline

**Supplementary Table 2.** The numbers (proportions, %) of the participants according to the change status of the MetS and its components by sex or age groups

**Supplementary Table 3.** Hazard risk of kidney stones presence with risk of incident cardiovascular events by status changes in metabolic syndrome from baseline to follow-up in women

**Supplementary Table 4.** Hazard risk of kidney stones presence with risk of incident cardiovascular events by status changes in metabolic syndrome from baseline to follow-up in men

**Supplementary Table 5.** Association of kidney stones with risk of longitudinal cardiovascular profiles

**Supplementary Figure 1**

**
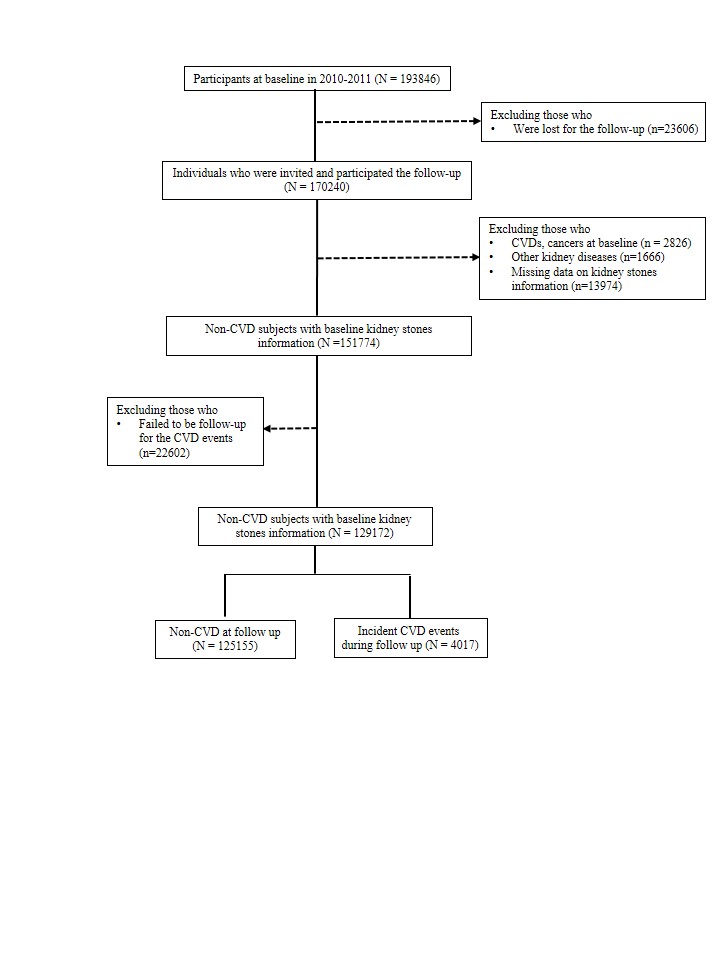
**

**Supplementary Figure 2**

**A.**

**B.**

**Supplementary Figure 3**

**
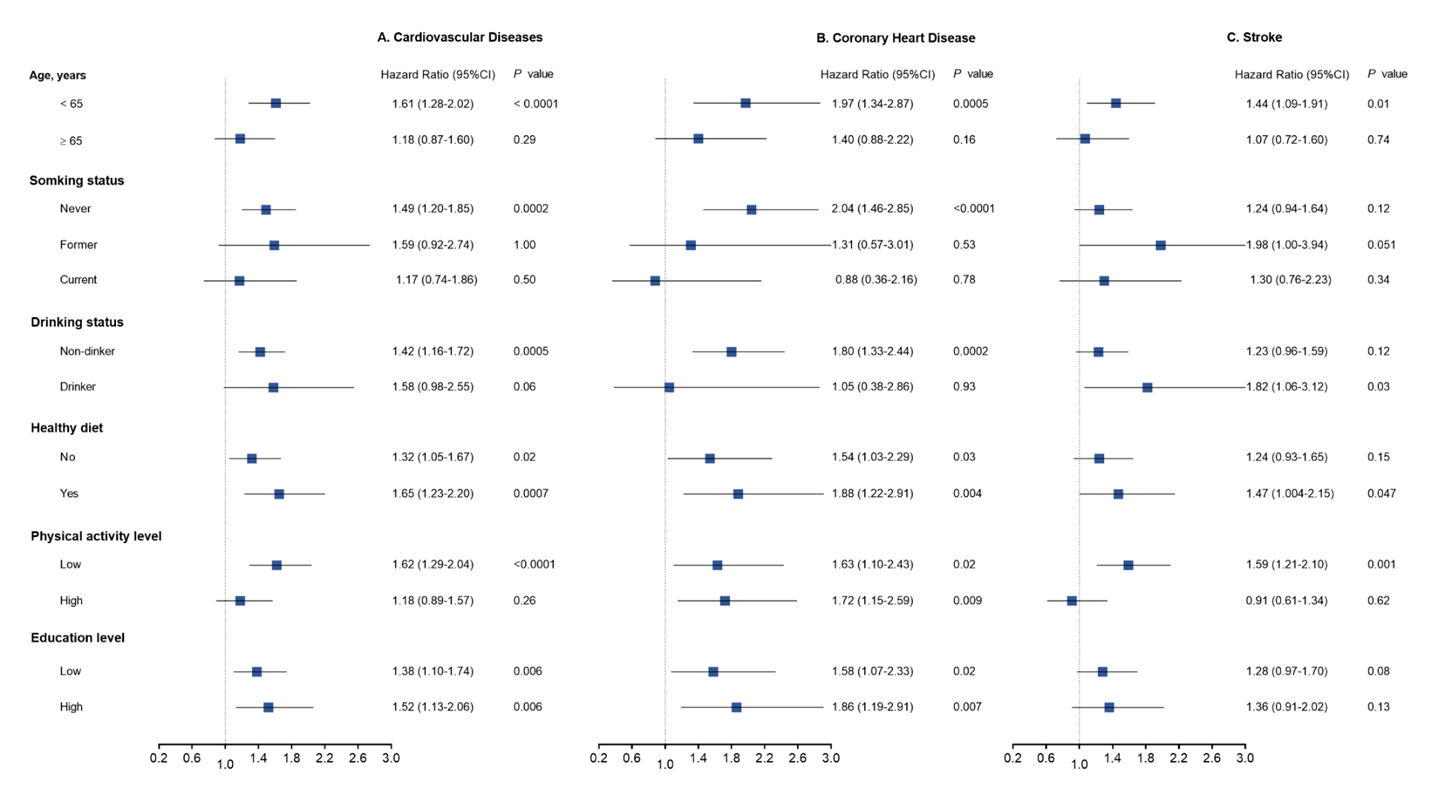
**

**Supplementary Table 1.** Stratified analysis of the hazard risk of kidney stones with a risk of incident cardiovascular events by metabolic syndrome (MetS) status at baseline

|  | **Cardiovascular Diseases, CVD** | | | **Coronary Heart Disease, CHD** | | | **Stroke** | | |
| --- | --- | --- | --- | --- | --- | --- | --- | --- | --- |
|  | **HR (95% CI)** | ***P* value** | ***P*-inter** | **HR (95% CI)** | ***P* value** | ***P*-inter** | **HR (95% CI)** | ***P* value** | ***P*-inter** |
| **Metabolic syndrome, no** |  |  | 0.0006 |  |  | 0.06 |  |  | 0.002 |
| Person-year | 294169 |  |  | 295495 |  |  | 294583 |  |  |
| No. of case | 1662 |  |  | 617 |  |  | 1074 |  |  |
| Incidence rate, % | 2.44 |  |  | 0.90 |  |  | 1.58 |  |  |
|  | 1.12 (0.80-1.56) | 0.52 |  | 1.29 (0.76-2.16) | 0.34 |  | 1.00 (0.64-1.54) | 1.00 |  |
| **Metabolic syndrome, yes** |  |  |  |  |  |  |  |  |  |
| Person-year | 252033 |  |  | 254002 |  |  | 252602 |  |  |
| No. of case | 2212 |  |  | 582 |  |  | 1521 |  |  |
| Incidence rate, % | 3.84 |  |  | 1.28 |  |  | 2.64 |  |  |
|  | 1.66 (1.33-2.06) | <0.0001 |  | 2.02 (1.41-2.88) | 0.0001 |  | 1.50 (1.14-1.96) | 0.003 |  |
|  |  |  |  |  |  |  |  |  |  |
| **Non-central obesity** |  |  | 0.52 |  |  | 0.79 |  |  | 0.64 |
| Person-year | 256383 |  |  | 257712 |  |  | 256780 |  |  |
| No. of case | 1571 |  |  | 575 |  |  | 1021 |  |  |
| Incidence rate, % | 2.67 |  |  | 0.98 |  |  | 1.73 |  |  |
|  | 1.37 (1.02-1.84) | 0.04 |  | 1.69 (1.06-2.69) | 0.03 |  | 1.25 (0.86-1.83) | 0.25 |  |
| **Central obesity** |  |  |  |  |  |  |  |  |  |
| Person-year | 312996 |  |  | 315123 |  |  | 313649 |  |  |
| No. of case | 2357 |  |  | 798 |  |  | 1608 |  |  |
| Incidence rate, % | 3.45 |  |  | 1.17 |  |  | 2.36 |  |  |
|  | 1.50 (1.19-1.88) | 0.0006 |  | 1.73 (119-2.52) | 0.004 |  | 1.36 (1.02-1.81) | 0.04 |  |
|  |  |  |  |  |  |  |  |  |  |
| **Non-diabetes** |  |  | 0.86 |  |  | 0.28 |  |  | 0.56 |
| Person-year | 441051 |  |  | 443236 |  |  | 441700 |  |  |
| No. of case | 2465 |  |  | 836 |  |  | 1677 |  |  |
| Incidence rate, % | 2.50 |  |  | 0.85 |  |  | 1.70 |  |  |
|  | 1.46 (1.14-1.86) | 0.002 |  | 1.49 (0.97-2.28) | 0.07 |  | 1.44 (1.081.93) | 0.01 |  |
| **Diabetes** |  |  |  |  |  |  |  |  |  |
| Person-year | 136289 |  |  | 137603 |  |  | 136714 |  |  |
| No. of case | 1537 |  |  | 573 |  |  | 994 |  |  |
| Incidence rate, % | 5.10 |  |  | 1.90 |  |  | 3.30 |  |  |
|  | 1.40 (1.06-1.84 | 0.02 |  | 1.94 (1.30-2.89) | 0.001 |  | 1.12 (0.77-1.63) | 0.55 |  |
|  |  |  |  |  |  |  |  |  |  |
| **Non-hypertension** |  |  | 0.74 |  |  | 0.89 |  |  | 0.76 |
| Person-year | 336017 |  |  | 337114 |  |  | 336437 |  |  |
| No. of case | 1327 |  |  | 529 |  |  | 818 |  |  |
| Incidence rate, % | 1.78 |  |  | 0.71 |  |  | 1.10 |  |  |
|  | 1.20 (0.85-1.69) | 0.30 |  | 1.05 (0.58-1.92) | 0.87 |  | 1.26 (0.83-1.91) | 0.28 |  |
| **Hypertension** |  |  |  |  |  |  |  |  |  |
| Person-year | 240284 |  |  | 242692 |  |  | 240930 |  |  |
| No. of case | 2672 |  |  | 874 |  |  | 1855 |  |  |
| Incidence rate, % | 4.95 |  |  | 1.62 |  |  | 3.44 |  |  |
|  | 1.54 (1.24-1.90) | <0.0001 |  | 2.07 (1.48-2.89) | < 0.0001 |  | 1.31 (1.00-1.73) | 0.05 |  |
|  |  |  |  |  |  |  |  |  |  |
| **Dyslipidemia, no** |  |  | 0.64 |  |  | 0.19 |  |  | 0.77 |
| Person-year | 333204 |  |  | 335041 |  |  | 333726 |  |  |
| No. of case | 2168 |  |  | 748 |  |  | 1458 |  |  |
| Incidence rate, % | 2.87 |  |  | 1.00 |  |  | 1.92 |  |  |
|  | 1.38 (1.06-1.80) | 0.02 |  | 1.34 (0.82-2.18) | 0.24 |  | 1.36 (0.99-1.88) | 0.06 |  |
| **Dyslipidemia, yes** |  |  |  |  |  |  |  |  |  |
| Person-year | 227418 |  |  | 228953 |  |  | 227911 |  |  |
| No. of case | 1750 |  |  | 621 |  |  | 1165 |  |  |
| Incidence rate, % | 3.46 |  |  | 1.23 |  |  | 2.31 |  |  |
|  | 1.49 (1.17-1.91) | 0.002 |  | 2.02 (1.40-2.92) | 0.0002 |  | 1.26 (0.91-1.75) | 0.17 |  |
|  |  |  |  |  |  |  |  |  |  |
| **eGFR, ≥ 90** ml/min per 1.73m^2^ |  |  | 0.21 |  |  | 0.36 |  |  | 0.40 |
| Person-year | 402820 |  |  | 404847 |  |  | 403380 |  |  |
| No. of case | 2079 |  |  | 654 |  |  | 1468 |  |  |
| Incidence rate, % | 2.32 |  |  | 0.73 |  |  | 1.64 |  |  |
|  | 1.29 (0.99-1.70) | 0.06 |  | 1.46 (0.91-2.35) | 0.12 |  | 1.20 (0.86-1.67) | 0.06 |  |
| **eGFR, < 90** ml/min per 1.73m^2^ |  |  |  |  |  |  |  |  |  |
| Person-year | 174785 |  |  | 176256 |  |  | 175293 |  |  |
| No. of case | 1915 |  |  | 750 |  |  | 1200 |  |  |
| Incidence rate, % | 4.89 |  |  | 1.91 |  |  | 3.06 |  |  |
|  | 1.59 (1.25-2.04) | 0.0002 |  | 1.96 (1.35-2.85) | 0.0003 |  | 1.41 (1.02-1.94) | 0.04 |  |

Data are hazard ratio (HR), 95% confidence interval (CI). *P* values were calculated from the Cox regression models. The results were adjusted for age (years), sex, body mass index (kg/m2), waist circumference (cm), quartiles of physical activity, quartiles of sedentary time, smoking status (current, former, and never), alcohol drinking (g/day), and education level (high school and above, or less), systolic blood pressure (mmHg), diastolic blood pressure (mmHg), fasting plasma glucose (mmol/L), oral glucose tolerance test (OGTT) 2-hour glucose (mmol/L), HOMA-IR, low-density lipoprotein (LDL) cholesterol (mmol/l), high-density lipoprotein (HDL) cholesterol (mmol/l), triglycerides (mmol/L), eGFR, dietary score, and gall stone (yes or no). P for interaction were tested from the multivariable Cox regression models with kidney stones, each stratification variable, the multiplicative interaction term, and the co-variables as above in the same models simultaneously.

**Supplementary Table 2.** The numbers (proportions, %) of the participants according to the change status of the MetS and its components by sex or age groups.

|  | Numbers of the participants (proportions, %) | |
| --- | --- | --- |
|  | Men | Women |
| **Mets status change** | | |
| Remain no MetS | 14285 (46.6) | 22293 (37.3) |
| Incident MetS | 4075 (13.3) | 8183 (13.7) |
| MetS remission | 3561 (11.6) | 6536 (10.9) |
| Sustained MetS | 8719 (28.5) | 22721 (38.0) |
| **Central obesity status changes** | | |
| Remain no central obesity | 15482 (49.6) | 14848 (24.4) |
| Incident central obesity | 4120 (13.2) | 8579 (14.1) |
| Central obesity remission | 2603 (8.33) | 5549 (9.13) |
| Sustained central obesity | 9027 (28.9) | 31810 (52.3) |
| **Triglycerides metabolism status changes** | | |
| Remain normal triglycerides | 18005 (53.4) | 34639 (53.4) |
| Incident high triglycerides | 4213 (12.5) | 9628 (14.9) |
| Triglycerides metabolism remission | 3635 (10.8) | 6439 (9.93) |
| Sustained high triglycerides | 7840 (23.3) | 14114 (21.8) |
| **HDL_c metabolism status changes** | | |
| Remain normal HDL_c | 22954 (68.0) | 26876 (41.4) |
| Incident low HDL_c | 3174 (9.41) | 9371 (14.5) |
| HDL_c metabolism remission | 4155 (12.3) | 9400 (14.5) |
| Sustained low HDL_c | 3460 (10.3) | 19204 (29.6) |
| **Blood pressure status changes** | | |
| Remain normal blood pressure | 5879 (18.3) | 17563 (28.3) |
| Incident high blood pressure | 3801 (11.8) | 761 (12.3) |
| High blood pressure remission | 3192 (9.94) | 6168 (9.94) |
| Sustained high blood pressure | 19251 (59.9) | 30684 (61.5) |
| **Glucose metabolism status changes** | | |
| Remain normal blood glucose | 10233 (29.9) | 24825 (38.0) |
| Incident high blood glucose | 4493 (13.1) | 8228 (12.6) |
| Glucose metabolism remission | 4681 (13.7) | 9188 (14.1) |
| Sustained high blood glucose | 14789 (43.3) | 23094 (35.4) |

**Supplementary Table 3.** Hazard risk of kidney stones presence with risk of incident cardiovascular events by status changes in metabolic syndrome from baseline to follow-up in women.

|  | **Cardiovascular Diseases** | | **Coronary Heart Disease** | | **Stroke** | |
| --- | --- | --- | --- | --- | --- | --- |
|  | **HR (95% CI)** | ***P* value** | **HR (95% CI)** | ***P* value** | **HR (95% CI)** | ***P* value** |
| **MetS status changes** |  |  |  |  |  |  |
| Remain no MetS | 1.84 (0.86-3.93) | 0.12 | 1.34 (0.32-5.58) | 0.68 | 2.07 (0.84-5.10) | 0.11 |
| Incident MetS | 2.49 (1.08-5.71) | 0.03 | 5.06 (1.49-17.2) | 0.009 | 1.59 (0.50-5.06) | 0.44 |
| MetS remission | 0.50 (0.07-3.59) | 0.49 | 0 | 0.99 | 0.64 (0.09-4.64) | 0.66 |
| Sustained MetS | 2.22 (1.50-3.29) | <0.0001 | 3.94 (2.10-7.39) | <0.0001 | 1.73 (1.06-2.83) | 0.03 |
| **Central obesity status changes** |  |  |  |  |  |  |
| Remain no central obesity | 1.10 (0.35-3.49) | 0.87 | 2.90 (0.65-12.9) | 0.16 | 0.49 (0.07-3.57) | 0.48 |
| Incident central obesity | 3.63 (1.57-8.35) | 0.002 | 4.79 (1.11-20.7) | 0.04 | 3.95 (1.58-9.89) | 0.003 |
| Central obesity remission | 2.06 (0.64-6.65) | 0.22 | 0 | 0.99 | 2.77 (0.84-9.10) | 0.09 |
| Sustained central obesity | 1.97 (1.36-2.86) | 0.0004 | 3.17 (1.74-5.75) | 0.0002 | 1.51 (0.94-2.43) | 0.09 |
| **Triglycerides metabolism status changes** |  |  |  |  |  |  |
| Remain normal triglycerides | 2.03 (1.29-3.18) | 0.002 | 2.49 (1.15-5.40) | 0.02 | 1.75 (1.00-3.07) | 0.048 |
| Incident high triglycerides | 1.54 (0.57-4.20) | 0.40 | 5.05 (1.15-22.2) | 0.03 | 0.87 (0.22-3.56) | 0.85 |
| Triglycerides metabolism remission | 2.13 (0.86-5.29) | 0.10 | 4.36 (1.27-15.1) | 0.02 | 1.76 (0.55-5.63) | 0.34 |
| Sustained high triglycerides | 2.03 (1.12-3.66) | 0.02 | 2.69 (0.95-7.60) | 0.06 | 1.80 (0.88-3.70) | 0.11 |
| **HDL_c metabolism status changes** |  |  |  |  |  |  |
| Remain normal HDL_c | 0.78 (0.32-1.90) | 0.59 | 1.54 (0.47-4.90) | 0.48 | 0.44 (0.11-1.79) | 0.25 |
| Incident low HDL_c | 2.48 (1.21-5.08) | 0.01 | 5.03 (1.74-14.5) | 0.003 | 1.60 (0.59-4.38) | 0.36 |
| HDL_c metabolism remission | 2.70 (1.24-5.87) | 0.01 | 1.54 (0.20-11.7) | 0.67 | 2.84 (1.22-6.61) | 0.02 |
| Sustained low HDL_c | 2.54 (1.64-3.95) | <0.0001 | 3.76 (1.80-7.85) | 0.0004 | 2.19 (1.29-3.74) | 0.004 |
| **Blood pressure status changes** |  |  |  |  |  |  |
| Remain normal blood pressure | 2.23 (0.97-5.13) | 0.06 | 2.30 (0.54-9.71) | 0.26 | 2.16 (0.78-5.95) | 0.14 |
| Incident high blood pressure | 0.86 (0.21-3.51) | 0.83 | 0 | 0.99 | 1.32 (0.32-5.44) | 0.71 |
| High blood pressure remission | 1.94 (0.46-8.11) | 0.36 | 0 | 0.99 | 3.21 (0.74-13.9) | 0.12 |
| Sustained high blood pressure | 2.11 (1.47-3.04) | 0.0001 | 4.32 (2.46-7.58) | <0.0001 | 1.53 (0.96-2.44) | 0.07 |
| **Glucose metabolism status changes** |  |  |  |  |  |  |
| Remain normal blood glucose | 2.36 (1.31-4.24) | 0.004 | 2.82 (1.02-7.82) | 0.046 | 2.33 (1.18-4.58) | 0.01 |
| Incident high blood glucose | 1.85 (0.75-4.56) | 0.18 | 5.62 (1.61-19.6) | 0.007 | 0.89 (0.22-3.63) | 0.87 |
| Glucose metabolism remission | 0.99 (0.24-4.05) | 0.98 | 1.76 (0.23-13.6) | 0.59 | 0.70 (0.10-5.08) | 0.72 |
| Sustained high blood glucose | 1.75 (1.16-2.65) | 0.008 | 2.10 (1.02-4.37) | 0.04 | 1.52 (0.92-2.52) | 0.10 |
| Data are hazard ratio (HR), 95% confidence interval (CI). *P* values were calculated from the Cox regression models. The adjustments included age (year), sex, body mass index (kg/m^2^), waist circumference (cm), quartiles of physical activity, quartiles of sedentary time, smoking status (current, former, and never), alcohol drinking (g/day), and education level (high school and above, or less), systolic and diastolic blood pressure (mmHg), fasting plasma glucose (mmol/L), oral glucose tolerance test (OGTT) 2-hour glucose (mmol/L), HOMA-IR, low- and high-density lipoprotein cholesterol (mmol/L), triglycerides (mmol/L), gall stone (yes or no), diet score, and eGFR. | | | | | | |

**Supplementary Table 4.** Hazard risk of kidney stones presence with risk of incident cardiovascular events by status changes in metabolic syndrome from baseline to follow-up **in men**.

|  | **Cardiovascular Diseases** | | **Coronary Heart Disease** | | **Stroke** | |
| --- | --- | --- | --- | --- | --- | --- |
|  | **HR (95% CI)** | ***P* value** | **HR (95% CI)** | ***P* value** | **HR (95% CI)** | ***P* value** |
| **MetS status changes** |  |  |  |  |  |  |
| Remain no MetS | 1.06 (0.52-2.18) | 0.87 | 2.06 (0.80-5.27) | 0.13 | 0.56 (0.18-1.78) | 0.33 |
| Incident MetS | 2.34 (0.99-5.51) | 0.05 | 3.18 (0.69-14.7) | 0.14 | 2.07 (0.73-5.87) | 0.17 |
| MetS remission | 2.45 (1.20-5.02) | 0.01 | 3.28 (0.72-15.1) | 0.13 | 2.26 (1.00-5.11) | 0.05 |
| Sustained MetS | 0.86 (0.44-1.68) | 0.66 | 0.62 (0.15-2.55) | 0.51 | 0.95 (0.45-2.04) | 0.90 |
| **Central obesity status changes** |  |  |  |  |  |  |
| Remain no central obesity | 1.51 (0.87-2.61) | 0.14 | 2.38 (1.01-5.57) | 0.046 | 1.17 (0.57-2.40) | 0.67 |
| Incident central obesity | 2.24 (1.01-4.95) | 0.05 | 2.77 (0.63-12.3) | 0.18 | 2.06 (0.81-5.25) | 0.13 |
| Central obesity remission | 0.94 (0.29-3.03) | 0.92 | 0 | 0.99 | 1.47 (0.45-4.85) | 0.53 |
| Sustained central obesity | 0.99 (0.51-1.93) | 0.97 | 1.08 (0.34-3.48) | 0.90 | 0.85 (0.34-2.08) | 0.71 |
| **Triglycerides metabolism status changes** |  |  |  |  |  |  |
| Remain normal triglycerides | 1.60 (1.02-2.50) | 0.04 | 2.59 (1.29-5.19) | 0.007 | 1.19 (0.67-2.14) | 0.55 |
| Incident high triglycerides | 0.29 (0.04-2.13) | 0.23 | 0 | 0.99 | 0.38 (0.05-2.82) | 0.35 |
| Triglycerides metabolism remission | 1.19 (0.47-3.02) | 0.71 | 0.76 (0.10-5.79) | 0.79 | 1.42 (0.49-4.07) | 0.52 |
| Sustained high triglycerides | 1.20 (0.55-2.61) | 0.64 | 0.83 (0.19-3.59) | 0.81 | 1.58 (0.67-3.72) | 0.30 |
| **HDL_c metabolism status changes** |  |  |  |  |  |  |
| Remain normal HDL_c | 1.18 (0.71-1.96) | 0.52 | 1.25 (0.50-3.13) | 0.63 | 1.18 (0.66-2.12) | 0.58 |
| Incident low HDL_c | 1.52 (0.69-3.33) | 0.30 | 1.62 (0.49-5.38) | 0.43 | 1.43 (0.51-4.04) | 0.50 |
| HDL_c metabolism remission | 1.74 (0.74-4.11) | 0.21 | 2.90 (0.81-10.4) | 0.10 | 1.25 (0.38-4.10) | 0.71 |
| Sustained low HDL_c | 1.17 (0.47-2.93) | 0.73 | 0.68 (0.09-5.06) | 0.70 | 1.43 (0.51-4.02) | 0.49 |
| **Blood pressure status changes** |  |  |  |  |  |  |
| Remain normal blood pressure | 2.00 (0.61-6.54) | 0.25 | 1.64 (0.21-12.8) | 0.64 | 2.15 (0.50-9.25) | 0.30 |
| Incident high blood pressure | 0.71 (0.10-5.31) | 0.74 | 3.21 (0.39-26.3) | 0.28 | 0 | 0.99 |
| High blood pressure remission | 1.38 (0.42-4.51) | 0.59 | 2.33 (0.53-10.3) | 0.26 | 0.77 (0.10-5.81) | 0.80 |
| Sustained high blood pressure | 1.39 (0.94-2.06) | 0.10 | 1.38 (0.67-2.85) | 0.38 | 1.41 (0.89-2.21) | 0.15 |
| **Glucose metabolism status changes** |  |  |  |  |  |  |
| Remain normal blood glucose | 1.45 (0.75-2.77) | 0.27 | 2.54 (0.98-6.60) | 0.06 | 0.98 (0.40-2.44) | 0.97 |
| Incident high blood glucose | 2.06 (0.81-5.28) | 0.13 | 2.56 (0.53-12.4) | 0.24 | 1.76 (0.53-5.84) | 0.35 |
| Glucose metabolism remission | 2.17 (0.84-5.56) | 0.11 | 3.41 (0.75-15.5) | 0.11 | 1.72 (0.51-5.77) | 0.38 |
| Sustained high blood glucose | 0.95 (0.57-1.60) | 0.86 | 0.57 (0.18-1.80) | 0.34 | 1.20 (0.68-2.10) | 0.53 |

Data are hazard ratio (HR), 95% confidence interval (CI). *P* values were calculated from the Cox regression models. The adjustments included age (year), sex, body mass index (kg/m^2^), waist circumference (cm), quartiles of physical activity, quartiles of sedentary time, smoking status (current, former), alcohol drinking (g/day), and education level (high school and above, or less), systolic and diastolic blood pressure (mmHg), fasting plasma glucose (mmol/L), oral glucose tolerance test (OGTT) 2-hour glucose (mmol/L), HOMA-IR, low- and high-density lipoprotein cholesterol (mmol/L), triglycerides (mmol/L), gall stone (yes or no), diet score, and eGFR.

**Supplementary Table 5.** Association of kidney stones with risk of longitudinal cardiovascular profiles

|  | Model 1 | | Model 2 | |
| --- | --- | --- | --- | --- |
|  | Beta ± SE | *P_1_* value | Beta ± SE | *P_2_* value |
| Blood pressure |  |  |  |  |
| Systolic blood pressure (mmHg) | -0.527 ± 0.318 | 0.097 | -0.420 ± 0.326 | 0.197 |
| Diastolic blood pressure (mmHg) | 0.180 ± 0.188 | 0.339 | 0.103 ± 0.195 | 0.596 |
| Glucose metabolism |  |  |  |  |
| Fasting plasma glucose (mmol/L) | -0.007 ± 0.010 | 0.524 | 0.110 ± 0.034 | 0.001 |
| Fasting serum insulin (mmol/L) | 0.048 ± 0.009 | <.0001 | 0.025 ± 0.0001 | 0.003 |
| HOMA-IR | 0.044 ± 0.010 | <0.0001 | 0.020 ± 0.009 | 0.032 |
| Lipid propile |  |  |  |  |
| Low-density lipoprotein cholesterol (mmol/L) | 0.029 ± 0.015 | 0.048 | 0.034± 0.016 | 0.031 |
| High-density lipoprotein cholesterol (mmol/L) | -0.015 ± 0.006 | 0.011 | -0.013 ± 0.006 | 0.025 |
| Triglycerides (mmol/L) | 0.035 ± 0.021 | 0.094 | 0.0001 ± 0.023 | 0.953 |
| Total cholesterol (mmol/L) | -0.008 ± 0.042 | 0.843 | -0.056± 0.080 | 0.489 |

Data were presented as Estimate (Beta) ± Standard Error (SE).

Model 1, age (years) and sex adjusted.

Model 2, further adjusted body mass index (kg/m^2^), waist circumference (cm), quartiles of physical activity, quartiles of sedentary time, smoking status (current, former and never), alcohol drinking (g/day), education level (high school and above, or less), gall stone (yes or no), systolic and diastolic blood pressure (mmHg), low- and high-density lipoprotein cholesterol (mmol/L), triglycerides (mmol/L), diet score, and eGFR.
